# Effects of two commonly and limited used chemosterilants on* Lucilia sericata* egg surface sterilization

**DOI:** 10.1007/s00436-025-08515-y

**Published:** 2025-06-27

**Authors:** Nevra Polat, Salih Mollahaliloglu, Murat Koc

**Affiliations:** 1 Department of Traditional Complementary and Integrative Medicine, Institute of Public Health, Ankara Yıldırım Beyazıt University, Ankara, Turkey; 2 Department of Internal Medical Sciences, Faculty of Medicine, Ankara Yıldırım Beyazıt University, Ankara, Turkey

**Keywords:** Maggot debridement therapy, *Lucilia sericata*, Egg sterilization

## Abstract

Maggot therapy (MT) is the most common medical use of sterile fly larvae of *Lucilia sericata* and other species of the family Calliphoridae as an alternative to surgery and long-term antiseptic therapy in the treatment of deep and infected non-healing wounds. Effective and reliable MT requires an aseptic technique to prevent bacterial infection of the wound. However, due to the habitat of Calliphoridae flies, the outer surface of their eggs is often contaminated with bacteria. In this study, *Lucilia sericata* eggs were sterilized using two techniques. NaOCI (sodium hypochlorite), which is widely used as a sterilant (Sterilant 1-S1), and Lysol surface disinfectant, which is rarely used (Sterilant 2-S2), and a control (unsterilized) were chosen. Sterilization efficacy, egg survival, and mortality were evaluated comparatively at 0.05, 1, 2, and 3 (%) concentrations and 1-, 2-, 3-, and 4-min application times at each concentration, and bacterial growth in the post-sterilization environment was controlled. In the in vitro sterility efficiency and reliability test, no bacterial colonies were found in sterilized eggs transferred to tryptic soy agar (TSA) culture. In contrast, bacterial and fungal growth was detected in unsterilized eggs. Protocol S1 provided effective egg surface sterilization at a concentration of 0.05% with a 1-min exposure time. This treatment resulted in 85.12% survival rate and 14.88% egg mortality. Based on its optimal balance of low mortality, high viability, and effective sterilization, we recommend a 1-min immersion in 0.05% NaOCl solution for sterilization of *Lucilia sericata* eggs.

## Introductıon

The maggot is the immature form or larva of a fly of the family Calliphoridae, order Diptera, and is particularly associated with Brachyceran flies. Examples of Brachyceran flies include house flies (*Musca domestica),* horse flies (*Stomoxys calcitrans*, Tabanidae), pig duny flies (*Scathophaga stercoraria*, Yellow Dung Fly, Hippoboscidae), and the green bottle fly *L. sericata* of the genus Lucilia (Diptera: Calliphoridae). This species attacks animal or human carcasses (Shin et al. 2021); like some other fly species, they are usually found in filthy and dirt-covered environments with rubbish, carrion, and decaying materials and can mechanically transfer many pathogens that cause infectious diseases to organisms (Wohlfahrt et al. 2020). It causes myiasis or facultative myiasis (larval infestation of tissues) in humans (Martinez-Rojano et al. 2020) and animals (Huang et al., 2020). Apart from its harmful effect on the organism, it is known to cause significant damage to the leather and wool industry annually in some countries. However, the larval characteristic of this fly species is also helpful. *L. sericata* belonging to the genus Lucilia (Diptera: Calliphoridae) is essential in medical, veterinary, and forensic medicine, especially in forensic entomology and entomotoxicology (Sandoval-Arias et al. 2020). It attacks stinking and decaying carcasses and can be used in corpse investigations, such as determining the time and place of death with its larval development time (Shin et al. 2021).

In medicine and veterinary medicine, *Lucilia sericata* larvae (maggots) are widely used, especially in healing non-healing soft tissue wounds (Sherman and Pechter 1988; Sherman et al., 1995; Wolff and Hansson 2005; Sherman et al. 2007; Firoozfar et al., 2011). This treatment, called Larval Debridement Therapy (LDT) or Maggot Debridement Therapy (MDT), is the application of live and sterile fly larvae to necrotic (dead) skin lesions and helps the intended wound healing. It can stimulate rapid debridement and effective healing, especially in chronically infected wounds and multiple wounds such as life-threatening diabetes, ischaemic wounds, leg ulcers from atherosclerosis, gangrene, pressure sores and burns (Steenvoorde and Jukema 2004; Wolff and Hansson 2005; Blake et al. 2007; Gottrup and Jorgensen 2011; Naik and Harding 2017). Recent studies have pointed out that maggot therapy may be more effective than other prescribed traditional methods in terms of not being financially burdensome, being practical and easy to apply, cleaning soft tissue and infected wounds, and preventing amputation (Masiero and Thyssen 2016; Figueroa et al. 2006).

For effective and reliable treatment and success, it is essential to use effective techniques to remove the eggs and obtain aseptic larval (maggot) cultures from them, as well as to select the suitable species that utilize only necrotic tissue in their feeding process in the wound (Marcondes 2006). The surface of the fly egg (Diptera) is often contaminated by contact with several pathogens due to its many putrefactive feeding behaviors and niches (Singh et al. 2015). These pathogens must be removed before immature products can be used for therapeutic purposes. For the use of maggots (larvae) in treatment, sterilizing eggs with sterilants prevents secondary bacterial contamination of the wound during the treatment process (Brundage et al. 2016). Calliphorid larvae rely on microbes to support their feeding and development, and hence suppliers of medical maggots sterilize only the egg stage. It is impossible to state these are sterile, either—externally this is possible but internally they may host bacteria/symbionts transmitted vertically.

In the past, the surface of the eggs of many insect species has been sterilized with antiseptic agents and disinfectants. Some of the currently recommended sterilizers for the sterilization of the surface of fly eggs (Diptera) are mercury chloride saturated alcohol, hydrochloric acid, ammonium salts, sodium chloride, sodium hypochlorite, formalin, formaldehyde, Lysol™, ethanol, chlorhexidine, chloramine solution, alkyldimethyl benzalkonium chloride, hexylresorcinol, copper sulfate, Clorox, and active ingredients in commercial bleaches (Mackerras and Freney 1933; Simmons 1934; Connell 1981; Sherman et al. 1996; Mumcuoglu et al. 2001; Mohd Masri et al. 2005; Wolff and Hansson 2005; Jaklič et al., 2008; Brundage et al. 2016; Dallavecchia et al., 2019).

This diversity of sterilants is mainly related to cost and the mechanisms by which they act to kill the growth or inhibit the colonization of pathogens. Therefore, the choice of sterilant should consider cost/benefit and a method with the highest survival rate and the most effective sterility for mass larvae production (Fleischmann 2004). Sodium hypochlorite (NaClO) sterilant with a chlorine content of 15% and a pH value > 12, which has an oxidant effect and changes in bacterial cell metabolism and irreversible enzyme inhibition as the primary mechanism of action, is a widely used sterilant for the sterilization of fly eggs (Diptera). Its widespread use is associated with its advantages, such as rapid action, efficacy against a wide range of microorganisms even at low concentrations, ease of preparation, dilution, and application, and low toxicity and cost (Paulino 1999). Lysol™ is one of the other sterilants used on a limited basis (Brundage et al. 2016). It was first discovered 1889 during the cholera epidemic and attracted attention with its high germicidal activity. Lysol™ contains benzalkonium chloride and hydrogen peroxide, compounds that make chloride effective as an antimicrobial agent and preservative but have acidic contents. However, alkalinity is dominant and has a pH between 8.5 and 11.5. The disinfectant also kills the coronavirus (SARS-C0V-2) approved in the USA during the Covid-19 pandemic. In vitro studies on viruses and bacteria neutralize 99.9% of viruses, bacteria, and fungi on surfaces in seconds and can prevent the proliferation and spread of bacteria in 72 h.

Although many techniques and formulas have been proposed for sterilization in studies, egg sterilization is the most common, reliable, and effective method (Echeverri et al. 2010). Among the factors that are available for an effective, trustworthy, and positive result in larval wound treatment, the use of an effective technique in egg sterilization to obtain sterile maggots (larvae) from opened eggs is the foremost important factor (Baer 1931; Sherman and Wyle, 1996). Practitioners are generally very cautious to state that larvae are sterile, as there is a strong tendency for larvae to carry symbionts that have been vertically transmitted. In the selection of the sterilant used in egg sterilization, the survival conditions of the larvae, rapid action, effect on growth due to feeding, the benefit versus low cost, effectiveness against pathogens at reduced concentrations, low toxicity, and ease of practical application were considered. In some studies on sterilization, it has been reported that the concentration of the sterilant used varies in relation to species-specific egg production and small or large scale of colony production, that it is not applied at a certain concentration percentage and that the application time also varies (Greenberg 1970; Mohd Masri et al. 2005).

Among the species used for Maggot Therapy, the most commonly used species (Diptera: Calliphoridae) is *Lucilia sericata.* Although research on external surface sterilization of Diptera eggs has been reported, the link between the efficacy and safety of sterilization and the viability of sterilized eggs is often lacking (Chatterjee et al. 2016) Few studies have addressed mortality associated with sterilization techniques. Therefore, in this study, sterilization procedures were evaluated by testing the sterilization of eggs to obtain sterile larvae of *Lucilia sericata* used in wound treatment by testing the commonly used sodium hypochlorite and the limited use of Lysol™ sterilants. These tests were conducted to determine which sterilant concentration time would effectively sterilize fly eggs but would not affect the survival of immatures.

## Materıal and method

### *Lucilia sericata* fly colony under laboratory conditions

The study was carried out in the entomology laboratory located on the edge of a wooded area in the Çubuk campus of Ankara Yıldırım Beyazıt University, Department of Traditional, Complementary and Integrative Medicine. The adult of *L. sericata*, also known as green bottle glass fly or green flesh fly, is metallic green, bluish green, and coppery green in color and 5–10 mm in size. The life stage of *L. sericata* includes egg, larva (three stages), pre-pupa/pupa and adult forms, and the life cycle lasts 16 days. Flies were kept in cages (40 cm × 40 cm × 40 cm) in an insectarium with 12 h light and 12 h dark photoperiod with ventilation and timer system of *L. sericata* adults under optimum fly rearing conditions with an average temperature of 23 ± 2 °C and relative humidity of 60 ± 10%. The cages were surrounded by a material made of polyurethane and very dense fine tulle (40 cm), which prevented the entry of other parasites. The number of adult individuals in each cage was between 300 and 500. *L. sericata* cages in the culture groups were fed 50–60 g of chicken liver with high protein content twice a week for egg laying of adult flies and water with powdered sugar and honey as a carbohydrate source for their rearing. The study was conducted between September 2024 and December 2024. Different generations of *L. sericata* fly colonies were fed with chicken liver and allowed to lay eggs on the liver. The eggs obtained were placed in glass jars and fed with the daily required amount of chicken liver to ensure the growth and pupation of the larvae. After the larvae had fully grown and reached the prepupa stage, excess chicken liver was removed from the containers, and wood shavings were added to the containers to pupate the prepupa larvae. The pupae were transferred to new cages until adult flies emerged.

### Collection of eggs

In fly cages with *L. sericata* colonies, chicken liver placed in plastic Petri dishes was kept in the cages for 3–8 h to allow the female flies to lay eggs, and the Petri dishes were removed within 2–3 h to prevent the eggs from drying out under normal conditions. Egg groups were transferred to a glass Petri dish, and eggs were collected using a fine paintbrush (size 0) and sorted individually into 50-ml falcon tubes. In the egg sterilization procedure, four different concentrations of sterilants S1 (sodium hypochlorite (NaOCl solution) and S2 (Lysol solution) were planned to be applied at four different times. Each application time was based on 400 eggs per concentration, 100 eggs for each application time. Therefore, four different concentrations and four different times for one sterilant were tested with a total of 1600 eggs.

### Experimental procedures

The experiment consisted of two parts: (1) a test study to determine the percentage of surviving larvae for both sterilants applied; (2) an in vitro sterility efficacy and safety test study on fly eggs sterilized and unsterilized with the selected sterilants.

### Sterilization tests

In this study, to obtain sterile maggots (larvae), after determining the sensitivity level of the eggs to sterilant, S1 (sodium hypochlorite (NaOCl solution), which is widely used for sterilization, and S2 (Lysol solution), which is limitedly used, were used at the determined concentrations. All analyses were performed in a biological safety cabinet. Considering the literature values, sterilant concentrations were determined as 0.05%, 1%, 2%, and 3%, and application times were 1–2–3–4 min for our study. Female *L. sericata* flies were reared in the laboratory under sterile conditions and allowed to lay eggs on a high protein chicken liver substrate. Eggs were collected at 8–48 h. Eggs adhered to each other were separated with distilled water. Experimental analyses were carried out in two replicates at each concentration (0.05%, 1, 2, 3%) and application time (1–2–3–4 min) and the results were recorded. After adding 35 ml of sodium hypochlorite (NaOCI) to the Falcon tubes at each concentration to be analyzed, they were shaken and the Falcon tubes were left half tilted at each application time for the eggs to settle to the bottom. After the top liquid was poured off, the eggs were filtered in a Buchner funnel and washed one to two times with sterile distilled water. The same procedure was repeated for Lysol sterilant. The number of experimental replicates was limited by laboratory rearing optimization requirements (temperature and humidity control) and the seasonal timing of study initiation.

### Control of the hatching rate of eggs

The sterile eggs obtained were transferred to a plate containing sterile Tryptic Soy Agar (TSA) medium in a 9-cm Petri dish. They were incubated under fly conditions (temperature, 23 ± 2 °C) for 24 and 48 h, after which the hatching rate of the eggs was reported by observation. The number of eggs and the percentage of eggs hatching was calculated.

### *In vitro* sterility efficacy and reliability test

Apart from the sterility concentration and application time test, the eggs of the females laying eggs in the same 1–2-h time period on the food left in the culture cages were selected to be used for the sterility efficacy test. Some of the eggs were sterilized and some were not sterilized in equal numbers. Sterilized and unsterilized eggs were incubated overnight in TSA medium. The secretions of sterile and non-sterile larvae emerging from the eggs were obtained and sterility was checked by inoculating 25 μl into Tryptic Soy Agar (TSA) medium with a sterile Pasteur pipette. The edges of the medium plates were sealed with adhesive tapes to allow air passage but prevent larval escape. In vitro sterility was checked for bacterial growth after 48 h of incubation at 35 °C in inoculated Petri dishes. This method was performed according to Mohd Masri et al. (2005), Barnes and Gennard (2013), Mumcuoğlu (2013), and Thyssen et al. (2013.

### Survival ability of larvae

The survival rates of larvae emerging from eggs treated with the selected sterilants were determined, and the positive or negative effects of sterilants were evaluated. After cleaning the surfaces of the eggs with each tested sterilant, sterilized eggs were transferred to the prepared TSA medium and unsterilized eggs were transferred to liver nutrient medium. At 23 ± 2 °C for 48 h, the eggs hatched and larval development was monitored. Survival was then determined by counting the surviving second-stage larvae using a wet, fine paintbrush. The number of first instar larvae was not considered due to their small size and delicate nature, ultimately leading to mortality risk during counting. The experiment was carried out twice. A single-factor analysis of variance (ANOVA) was performed to determine possible differences in egg laying rate and larval survival compared to each treatment. The appropriate test method from multiple comparison tests was used for this procedure. Data were analyzed using ANOVA, and *p* < 0.05 was considered significant.

## Results

### Egg hatching

The effectiveness of *L. sericata* egg surface sterilization was evaluated by examining the eggs that were able to hatch after sterilization. A total of 1600 eggs were sterilized with S1, with 400 eggs used for each different concentration of the sterilizing agent. After 24 and 48 h, the number of hatched eggs was 374 (23.37%) and 1362 (85.12%), respectively. In comparison, when 1600 eggs were sterilized with S2, 275 (20.30%) and 1214 (75.85%) eggs hatched at the same time intervals. In the non-sterilized control group, the hatching rates were 426 (26.60%) at 24 h and 1576 (98.5%) at 48 h. In the comparative assessment, it was observed that during the critical first 24 h, when egg mortality is most likely to occur, the chemically treated eggs did not exhibit a substantial loss compared to the control group.

### Development, survival, and sterilization results of Lucilia sericata eggs in different sterilants

The study determined that sodium hypochlorite and Lysol solutions tested at various concentrations and exposure times successfully achieved sterilization, though differences were observed in surviving individuals (Tables 1 and 2). Across all applications, *L. sericata* individuals exhibited higher survival rates in sodium hypochlorite solution (Table 1). Furthermore, both sterilants demonstrated effective sterilization even at low concentrations (0.05%) and with brief 1-min exposures. No bacterial contamination was detected in sterility controls.

**Table 1:** Individual repeated measures ANOVA table of *Lucilia sericata* percentage survival according to NaClO sterilant applied at different concentrations at different times, (2 replicates per treatment), ± standard deviations (± SD) (Bonferroni test (*p* < 0.05))

Group	Application time	*N*	Survivor Individual *X* ± ss	*F*	p*
**0.05%**	1 min	100	84.00 ± 1.12	224,048,563.1	*p* < 0.001*
	2 min	100	82.00 ± 1.07		
	3 min	100	79.99 ± 0.83		
	4 min	100	78.98 ± 0.89		
**1%**	1 min	100	77.00 ± 1.49	116,133,832.4	*p* < 0.001*
	2 min	100	72.00 ± 1.63		
	3 min	100	69.98 ± 1.41		
	4 min	100	60.98 ± 0.89		
**2%**	1 min	100	60.00 ± 0.81	114,693,816.4	*p* < 0.001*
	2 min	100	58.00 ± 0.9		
	3 min	100	52.99 ± 0.82		
	4 min	100	51.98 ± 0.95		
**3%**	1 min	100	50.00 ± 0.79	84,422,366.1	*p* < 0.001*
	2 min	100	46.00 ± 0.75		
	3 min	100	41.98 ± 0.95		
	4 min	100	35.98 ± 1.07		

*p < *0.0.05*; **Bonferroni

**Table 2:** Individual repeated measures ANOVA table of *Lucilia sericata* percentage survival according to Lysol sterilant applied at different concentrations at different time values (2 replicates per treatment, ± standard deviations (± SD), Bonferroni test (*p* < 0.05))

Group	Application time	*N*	Survivor Individual *X* ± ss	*F*	*p*
**0.05%**	1 min	100	79.00 ± 1.25	110,491,493.8	*p* < 0.001*
	2 min	100	75.00 ± 1.42		
	3 min	100	72.98 ± 1.2		
	4 min	100	70.98 ± 1.14		
**1%**	1 min	100	71.00 ± 2.18	137,038,389.7	*p* < 0.001*
	2 min	100	68.00 ± 1.63		
	3 min	100	63.98 ± 1.09		
	4 min	100	61.98 ± 1		
**2%**	1 min	100	64.00 ± 2.31	5,053,601.69	*p* < 0.001*
	2 min	100	54.00 ± 0.9		
	3 min	100	52.98 ± 1.11		
	4 min	100	46.98 ± 1.23		
**3%**	1 min	100	45.00 ± 0.79	66,626,464.96	*p* < 0.001*
	2 min	100	42.00 ± 0.75		
	3 min	100	37.98 ± 0.95		
	4 min	100	34.98 ± 1.23		

*p < *0.0.05*; **Bonferroni

Whether there is a significant difference between the hatching times of *Lucilia sericata* eggs according to NaClO sterilant applied at different concentrations at different time values was compared by one-way analysis of variance (ANOVA) in repeated measures, which is a parametric method. As a result of this analysis, the times between which there was a significant difference were compared with the Bonferroni method, one of the multiple comparison methods (Table 1).

A significant difference (*p* < 0.05) was obtained between the percentage of living individuals depending on the *Lucilia sericata* egg opening time according to NaClO sterilant between different times within all percentages. The difference was obtained at all time intervals for each percentage. For NaClO sterilant, the measurements decreased significantly from the 1 st minute to the 4th minute for each percentage.

Whether there is a significant difference between the opening times of *Lucilia sericata* eggs according to Lysol sterilant at different time values was compared with one-way analysis of variance (ANOVA) in repeated measures, which is a parametric method. As a result of this analysis, the times between which there was a significant difference were compared with the Bonferroni method, one of the multiple comparison methods (Table 2).

A significant difference (*p* < 0.05) was obtained between the percentage of living individuals depending on the *Lucilia sericata* egg opening time according to Lysol sterilant between different times within all percentages. The difference was obtained at all time intervals for each percentage. For Lysol sterilant, the measurements decreased significantly from the 1 st minute to the 4th minute for each percentage.

As a result of our experimental sterilant efficacy and safety study, sodium hypochlorite and Lysol solutions with different concentrations and application times were found to have effective and reliable sterilization. However, different results were found in the survival rates, although not significantly different.

### In vitro* sterility efficacy and reliability test*

In the study, as the results of bacterial and fungal growth controls in the in vitro sterilant efficacy and reliability test of Sodium hypochlorite and Lysol solutions with the secretions obtained from the larvae developing in the medium with sterile and non-sterile egg packs (Fig. 3), no bacterial growth was observed in the media containing the secretions obtained from the larvae developing with sterile egg packs, but contamination was detected with bacterial growth at different intensities in both sterilants in the media containing the secretions obtained from the larvae developing with non-sterile egg packs (Fig. 3). When compared according to sterilants, more bacterial growth was observed in the media containing secretions obtained from larvae developing with unsterile egg packs of Lysol sterilant than in the media containing secretions obtained from larvae developing with unsterile egg packs of sodium hypochlorite sterilant.

## Discussion

The use of *Lucilia sericata* fly larvae, known as surgical maggots due to the antibiotic resistance of bacteria, in debridement treatment has recently been used in surgery (Sherman et al. 2000; Jaklič et al., 2008). The most important consideration when using *Lucilia sericata* larvae for wound healing is the need for sterilization for the safety and efficacy of the process. Flies are frequently infected with pathogens due to the unsterile habitats in which they live, and neglect of sterilization during use can potentially lead to secondary bacterial contamination of wounds through the easy entry of these harmful bacterial strains into the wound, ultimately causing death (Baer 1931). Numerous researches and studies have been carried out on external surface sterilization of eggs with different sterilants at different concentrations and their effects on egg hatching time and rate (Connell 1981; Mohd Masri et al. 2005; Brundage et al. 2016; Limsopatham et al. 2017). Research reports show that the sterilant used varies depending on the sterilant concentrations. Some research results indicate that depending on the sterilant used, the sterilant has a lethal effect on the egg hatching or has little effect on the egg surface. Therefore, the choice of sterilant, concentration, and duration of treatment are important in terms of causing egg death or unfertilized egg laying (infertility) of females. Several methods exist to sterilize *Lucilia sericata* eggs, including chemical and radiation sterilization. Chemical sterilization involves the treatment of eggs with a chemical solution that kills pathogens on the outer surface of the eggs. This method prevents bacterial contamination while allowing the larvae developing from the eggs to retain their therapeutic properties (Table 3). Radiation sterilization involves exposing the eggs to ionizing radiation that kills bacteria and pathogens on the outer surface of the eggs. This method has the advantage that the larvae developing from the eggs can penetrate deep into the tissues and destroy all pathogens.

**Table 3:** Control of bacterial growth on the egg surface with sterilants applied according to the total hatch percentage at 24 and 48 h

Sterilants	Total number of egg examined	Number of hatched eggs after 24 h	Hatching rate after 24 h	Number of hatched eggs after 48 h	Hatching rate after 48 h	Bacterial growth
S1 (NaCIO,Sodyum hipoklorid)	1600	374	23.37%	1362	85.12%	–
S2 (Lysol, surface disinfectant)	1600	275	20.30%	1214	75.85%	**–**
Control	1600	426	26.60%	1576	98.5%	+

For egg sterilization of *Lucilia sericata* (Meigen, Diptera: Calliphoridae) flies, low-concentration mercuric chloride solutions (Baer 1931; Mackerras and Freney 1933), formalin (Simmons,^1934^), Lysol (Sherman and Tran, 1995; Sherman, 1996), formaldehyde, sodium hypochlorite (NaOCl), Clorox (Teich 1986; Mumcuoglu et al. 2001), ethanol (Brookes and Lawly, 1961), alkyl dimethyl benzalkonium chloride (Connell 1981), or UV light (Mohd Masri et al. 2005) have been investigated and evaluated in various studies. For example, in the *Lucilia cuprina* egg sterilization study, 3% Lysol, formalin, and formaldehyde disinfectants were used, and their effectiveness was evaluated by immersion method. It was reported that 3% Lysol sterilized 96.67% of the egg samples by dipping, which resulted in 31.84% egg death. Five percent of formalin dipping was the least effective, with 33.51% egg death and only 3.33% sterilization (Brundage et al. 2016). Maggot Debridement they suggested that 3% Lysol is recommended for sterilizing Calliphoridae eggs before the development of larvae for treatment (Brundage et al. 2016). The present study found that Lysol sterilized 79% (± 1.25%) of the egg samples at 0.05% concentration and 1-min application time by immersion method, and 21% egg mortality was determined. Surviving individuals and mortality rates for the concentrations and application times of both sterilant tests used for effective and reliable sterilization within the scope of our study are shown in Table 4.

**Table 4:** Percentage of mortality and survival as a result of sterilization of S1 (NaOCI, sodium hypochlorite) and S2 (Lysol) at 0.05%, 1%, 2%, and 3% concentrations at all-time applications

S1 (NaOCI, Sodium hipoklorid)	0.05%	1%	2%	3%
**Survival rate**	85.12%	69.25%	55.75%	43.80%
**Mortality rate**	14.88%	30.75%	44.75%	56.20%
**S2 (Lysol)**	**0.05%**	**1%**	**2%**	**3%**
**Survival rate**	79.61%	70.06%	57.30%	42.50%
**Mortality rate**	20.39%	29.94%	42.70%	57.50%

**Fig. 1:**
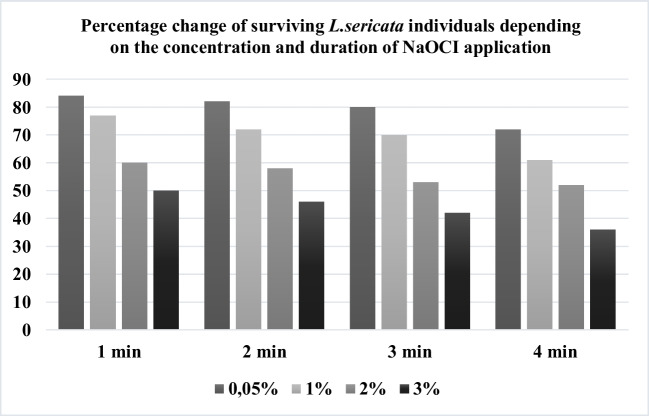
Survival of *Lucilia sericata* larvae hatched from eggs treated with different concentrations and time of NaCIO application

Thyssen et al. (2013) reported that there was no bacterial and pathogenicity growth in all application groups in in vitro bacterial and pathogenicity growth controls as a result of sterilization of the eggs of *Chrysomya megacephala* and *Chrysomya pretoria* fly species represented in the Calliphoridae family with NaOCl solution at 0.05% and 1% concentrations for two different application times of 1 and 3 min. However, it was reported that the rate of surviving individuals decreased by 1% 3-min applications. Our study determined that 1 min of NaOCl application at 0.05% concentration was sufficient for sterilizing *Lucilia sericata* eggs (Fig. 1). However, our findings that it is more ideal in terms of the number of surviving individuals compared to the other concentrations and application times applied and that it does not cause a significant loss in the population is in line with the research of Thyssen et al. (2013).

Dallavecchia, who investigated the effect of UV-C radiation on egg sterilization of *Calliphora vicina*, proved that after 12 min of egg exposure, the results showed that eggs were sterilized, and larval viability was 57%. However, in our current results, the hatchability was 75% and 85% (Dallavecchia et al., 2019).

**Fig. 2:**
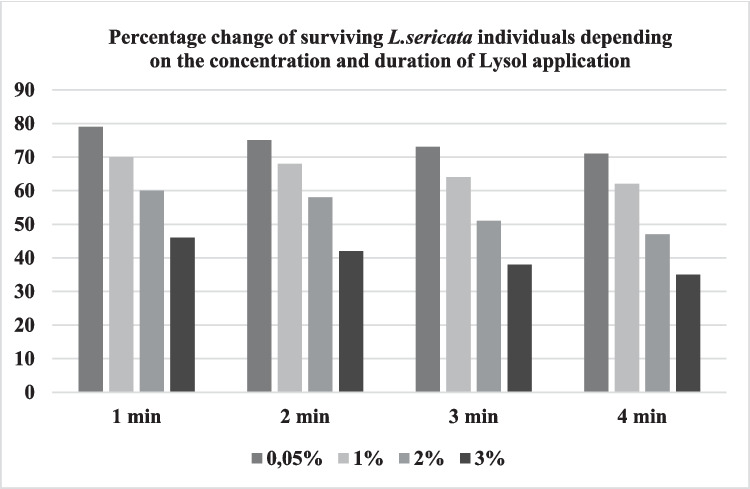
Survival of *Lucilia sericata* larvae hatched from eggs treated with different concentrations and time of Lysol application

Brundage et al. (2016) applied *Lucilia sericata* eggs using 5% formalin, 10% formalin, 3% Lysol, 5% H2CO, 1%, and 5% NaOCl sterilants at the concentrations determined by determining five different application times as 1, 3, 5, 7 and 10 min. They determined the egg eclosion increase rate and sterilant effectiveness of each sterilant on *Chrysomya rufifacies*, *Cochliomyia macellaria*, *Lucilia cuprina*, and *Lucilia sericata* species. According to the findings, 1% concentration NaOCl applied in 3 min provided moderate sterilizing efficiency, while 5% concentration NaOCl applied in 3 min provided high sterilizing efficiency but caused a decrease in egg eclosion and cracking of the chorion. Maisero and Thysen (2016) reported that the use of 1% sodium hypochlorite (NaClO) for the sterilization of *Cochliomyia macellaria* eggs demonstrated efficacy in an in vivo study focused on the treatment of diabetic wounds. According to the findings obtained in our study, 69.98% (± 1.41) of the egg samples survived in 1% concentration and 3 min NaOCl application, while 41.98% (± 0.95) of the egg samples survived in the highest application of 3% concentration and 3 min NaOCl application (Fig. 1). In Lysol sterilant 1% concentration and 3-min application, the rate of surviving individuals was 64% (± 1.09), while it was 38% (± 0.95) in 3% concentration and 3-min application (Fig. 2).

When it was evaluated whether there was a significant difference between the percentage of *Lucilia sericata* egg opening time and the percentage of surviving individuals according to NaOCl and Lysol sterilants applied at different concentrations at different time values, according to ANOVA Bonferroni method, a significant difference was obtained between the percentage of *Lucilia sericata* egg hatching time and the percentage of surviving individuals according to NaClO sterilant among the different times of all percentages (*p* < 0.05). Within the framework of the data obtained, we can say that NaClO sterilant, which is widely used, is ideal in terms of not causing loss with a concentration of 0.05%, 1-min application is sufficient for sterilization, and 84% (± 1.12) survival percentage; Lysol sterilant, which is not preferred, provides practical and reliable sterilization with a concentration of 0.05%, 1-min application is sufficient for sterilization and 79% (± 1.25) survival percentage.

**Fig. 3:**
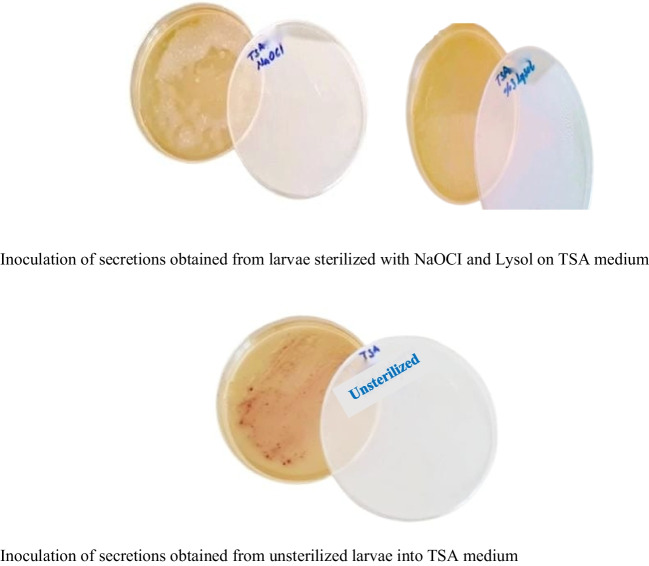

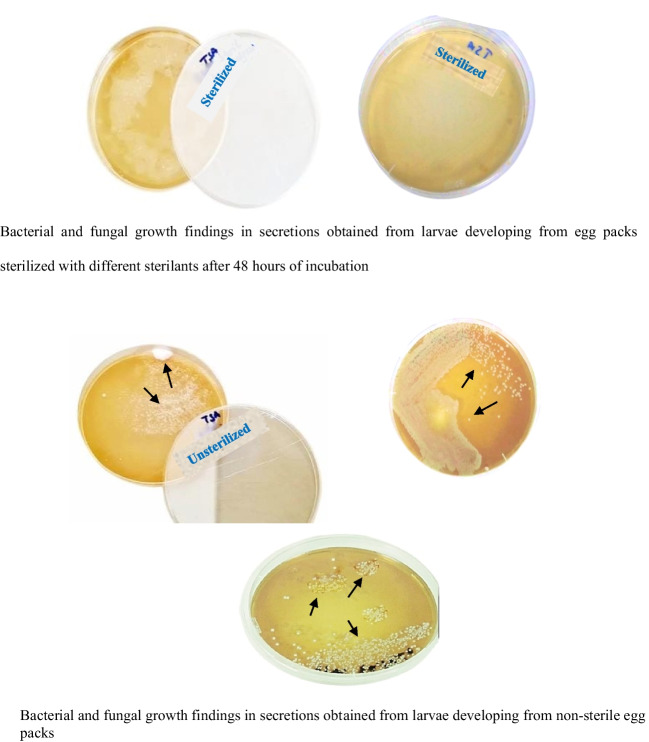
In vitro testing of the efficacy and reliability of NaOCI and Lysol for sterility

The results of the present study showed that NaOCI (sodium hypochlorite) and Lysol solutions applied at different concentrations and different application times effectively sterilized the outer surface of *L. sericata* eggs. No bacterial or fungal growth was found in the culture medium incubated for 48 h with the solution obtained from the outer egg (Fig. 3).

## Conclusion

Our research findings demonstrate that a 0.05% concentration of sterilant solution provides effective disinfection of eggs while preserving their viability after a 1-min application period. Notably, no decline in hatching rates was observed even with increased concentration and exposure time. Furthermore, the sterilants used were found to pose a low risk of inducing egg cracking or embryonic mortality. These results indicate that the proposed sterilization protocol achieves the high sterility level required for the medical use of larvae while simultaneously optimizing viability rates. A sterilization method for fly eggs that ensures minimal mortality while maximizing survival rates and sterility levels presents a significant advantage for the mass production of sterile larvae intended for medical use in wound therapy.

The surface solution derived from eggs sterilized with selected antiseptic agents demonstrated complete elimination of microorganisms (Gram-positive bacteria, Gram-negative bacteria, and yeast), with no bacterial or fungal colonies observed during in vitro microbiological testing. This finding provides a significant advantage for larvae developed from these eggs in the treatment of refractory wounds, as they remain completely sterile and free from harmful microorganisms, thereby preventing pathogenic contamination of wound sites. The absence of pathogenic microorganisms in larvae obtained from sterilized eggs substantially reduces infection risk in Maggot Debridement Therapy (MDT) while enhancing treatment biosafety. The efficacy and reliability of MDT are well-established through comprehensive microbiological and bacteriological testing protocols. Particularly in chronic wounds contaminated with antibiotic-resistant bacteria, microbiological confirmation of larval sterility through standardized testing is crucial for ensuring clinical success.

From a quality control perspective, the standardization of sterilization protocols and routine implementation of pathogenicity testing will be pivotal in production processes. Moreover, the combination of low mortality rates with high sterility and viability ratios enables cost-effective and sustainable industrial-scale production, which may significantly contribute to the broader adoption of medicinal larval therapy in clinical practice.

## Data Availability

No datasets were generated or analysed during the current study.
